# A psychometric evaluation of inter-professional education competency tool in nursing and medicine students

**DOI:** 10.1186/s12909-023-04991-x

**Published:** 2024-02-01

**Authors:** Davood Rasouli, Azam Norouzi, Ghobad Ramezani, Akram Hashemi

**Affiliations:** 1 Center for Educational Research in Medical Sciences (CERMS), Department of Medical Education, School of Medicine, Iran University of Medical Sciences (IUMS), Tehran, Iran; 2 Department of Medical Education, School of Medicine, Mashhad University of Medical Sciences, Mashhad, Iran; 3 Education Development Center, Kermanshah University of Medical Sciences, Kermanshah, Iran; 4 Medical Ethics Department, School of Medicine, Iran University of Medical Sciences (IUMS), Tehran, Iran

**Keywords:** Inter-professional collaboration, Inter-professional education, Evaluation, Inter-professional training, Psychometrics

## Abstract

**Introduction:**

Collaboration between nurses and doctors is necessary for offering care to patients. Using team performance assessment tools and surveying them can be effective in promoting inter-professional collaboration, and the lack of a credible tool to assess inter-professional collaboration competency between the two groups is a major challenge in the healthcare sector. The present study aimed to translate and conduct a psychometric investigation on the inter-professional education collaboration (IPEC) tool for the students of medicine and nursing.

**Methods:**

The present study was a cross-sectional one conducted as a psychometric investigation of the IPEC tool at the Iran University of Medical Sciences in 2022. The initial tool contained 42 items developed according to a 5-point Likert scale, which was translated into Persian with the consent of the original researcher. The validity index and the content validity ratio were investigated by a panel of 11 specialists in medical and clinical education, and its construct validity was evaluated using confirmatory factor analysis. Also, the second population of the study included medical and nursing students of Iran University of Medical Sciences and simple random sampling method. Moreover, the reliability of the instrument was investigated using internal consistency, Cronbach’s Alpha, and test–retest methods.

**Results:**

Based on the indicators calculated to perform a psychometric investigation over the above tool, it had acceptable reliability and validity according to the specialists. The tool evaluates inter-professional collaboration competency between the students of medicine and nursing across four areas (values and ethics, roles and responsibilities, inter-professional communication, and team-based care and teamwork). Moreover, Cronbach’s Alpha coefficient for the tool was determined at 0.84.

**Conclusion:**

The results of the study showed that the above tool could evaluate inter-professional competency as a valid and reliable questionnaire, and its results could be utilized in planning and education.

## Introduction

Doctors, nurses, and other professionals involved in healthcare services are typically educated separately, and, despite the importance of teamwork in the field, the majority of clinical units act as sets of distinct professions [1–4]. In a report published in 1973, the World Health Organization (WHO) uncovered medical students’ weaknesses in terms of working within healthcare teams and emphasized the adoption of an integrated approach in their education by recommending the need to merge various professional roles in the healthcare system [5]. Inter-professional education can enhance collaborative performance and the quality of the provided care [6, 7] and has attracted a lot of attention as an efficient approach that acts according to global changes [8]. Offering inter-professional education leads to the development of communicative skills, and this in turn improves trust among people, encourages them to discuss and share their views on matters concerning patients, elucidates individuals’ roles, and increases the possibility of offering better and more care to patients [9]. The attitudes and readiness of medical and nursing students to participate in inter-professional activities are the main predictors for the successful implementation of inter-professional education to develop joint action [10, 11]. Due to the revised approaches of healthcare systems arising from changes in demographic patterns, a load of illnesses, the increasing trend of chronic diseases, and the need to offer multilateral and complicated treatments, the need has arisen to direct educational approaches implemented for learners in healthcare disciplines toward inter-professional education [12]. In 2016, the IPEC committee was formed to approve the main competencies for inter-professional collaboration as a way of more conveniently merging approaches for population health in healthcare and the relevant professions and increasing collaboration to improve the results of both individual and population healthcare [13]. Studies have shown that inter-professional education and learning during medical and Nursing education bring about changes in learners’ knowledge and awareness about their roles and responsibilities toward one another and enhance teamwork and the participation of other professions [14–16].

Evaluating the practical competence of inter-professional jobs is highly prioritized in the case of students in healthcare fields. A self-report scale created to facilitate the competency-based evaluation of the IPE is the inter-professional Education Competence (IPEC) scale. The tool was first designed by Alan W. Dow et al. (2014) with 42 items across four areas [17]. As a way of promoting participatory approaches in a healthcare system, inter-professional education has to be considered from the early years of students’ education. Thus, a reliable and valid instrument is needed to follow up on educational programs and evaluate them. The instrument needs to be able to distinguish various demographic groups’ attitudes [18]. As a result, due to the importance of inter-professional education [19–21] and the lack of a valid and reliable instrument for that purpose in Iran, the study was carried out to translate and conduct a psychometric investigation of the IPEC tool for the students of medicine and Nursing.

This questionnaire is a relevant and useful tool with acceptable content validity and reliability, and it can be used to evaluate the competencies of interprofessional cooperation in medical and nursing students.

## Method

### Study design

This cross-sectional descriptive-survey study was designed to evaluate the psychometric properties of the IPEC instrument in medicine and nursing students at IUMS.

### Research sample ( Setting:)

The study was carried out at the Iran University of Medical Sciences and its subsidiary hospitals. The population included all experts and specialists in medical and clinical education, and the sample was selected using the stratified method. Thus, in the faculties of medicine and Nursing, the share of each faculty was determined according to the total sample size and the number of eligible students. Then, based on the number of eligible students inside each faculty, the sample was selected using the systematic random sampling technique out of 212 medical and nursing students (75 Nursing students and 136 medical students).

The study was conducted in four steps that are explained in detail below.

#### Stage 1: translation

After receiving consent from the designer of the IPEC instrument, it was translated based on the 4-stage protocol of the WHO. In the first step (also known as forward translation), instrument was translated in Persian language with an English proficient translator. In the second step, a panel of bilingual experts (in English and Persian) with adequate experience and familiarity with the terms was made up to detect and resolve any faulty terms and concepts in the translation. Then, the Persian translation was once again translated into English by another translator who did not know of the area covered by the scale. In the third step (pre-translation and cognitive interview), the scale was distributed among the target group (medicine and Nursing students). Briefing sessions were held to obtain the respondents familiar with the tool. In the sessions, in addition to investigating the responses to vague points in the translated scale, questions were asked about incomprehensible and unacceptable terms or their match with everyday language. Ultimately, a written report of the pre-test along with information concerning the participants’ cooperation was developed and used to prepare the final version of the translation.

#### Stage 2: content validity and steps

The preliminary version of the scale was handed over to 10 medical education specialists to investigate its qualitative content validity, and their views concerning relevance, comprehensibility, grammar, language, scoring, the main and major aspects of the investigated concept, the components and totality of the tool, the adequacy of the items, and their clarity and simplicity were consulted. Based on the collected opinions, areas that needed change were revised. Moreover, to investigate the quantitative validity of the tool, Strict’s content validity ratio (CVR_Strict_), the content validity index (CVI), and the modified Kappa statistic. Concerning each item, the CVR_Strict_ was calculated when necessary, and the CVI and modified Kappa were calculated based on relevance. The results of the CVI, CVR, and modified Kappa were judged using Ayre and Scally’s table, Waltz and Bausell index, and the views of Polit and Beck. Moreover, the total CVI of the tool was investigated using S-CVI_AVERAGE_.

#### Stage 3: construct validity (Exploratory and confirmatory factor analysis)

After data collection, first, the exploratory factor analysis was carried out in this stage. The data were extracted using the Principal Component Analysis (Varimax rotation). To determine the number of factors, eigenvalues above one and the scree plot were considered. The minimum factor loading was determined at 0.5, with no common factor loadings. Then, the ceiling and floor effects were investigated. In other words, the existence of the ceiling and floor effects in the instruments was checked based on the relative frequency of the samples with the highest and lowest attainable scores, respectively. The exploratory factor analysis was implemented to detect complicated patterns by discovering coherent data and testing the predictions. The method allows researchers to detect the principal aspects that match the theory considered in a certain study out of a relatively significant number of hidden constructs each of which is typically expressed with a list of items and can be reduced to a smaller number of common groups. On the other hand, confirmatory factor analysis is an attempt to approve research hypotheses and implement the path analysis chart as a way of explaining variables and factors.

In another sector, the construct validity of the instrument was carried out according to confirmatory factor analysis with PLS software. The Average Variance Extracted (AVE) is used to evaluate convergent validity in Smart PLS. The value of the coefficient varies from 0 to 1, and values above 0.5 are considered acceptable. Construct validity (CR), maximum shared variance (MSV), average shared variance (ASV), good-of-fit indicators (GFI), normed fit index (NFI), relative fit index (RFI), increasing fit index (IFI), comparative fit index (CFI), and the Root Mean Square Error of Approximation (RMSEA) were some other indicators calculated in this section.

#### Stage 4: reliability (Internal consistency and Stability)

The internal consistency of the total instrument (the final version) and its subscales (factors extracted during the factor analysis) was calculated across all investigated samples using Cronbach’s Alpha coefficient. In terms of stability, the reliability of the instrument was investigated using the test–retest method and intra-class correlation coefficient, while its internal consistency was investigated using Cronbach’s Alpha.

### Data analysis

The data were analyzed with SPSS V.16. The Kaiser–Meyer–Olkin (KMO)’s measure of sampling adequacy and Bartlett’s test of Sphericity were used to determine the factor ability of the sample and the fit of the factor analysis. A KMO value higher than 0.5 is acceptable. EFA was performed by principal component analysis followed by varimax rotation. Eigenvalues and factor loadings were considered higher than1 and 0.3, respectively. Then, the confirmatory factor analysis method was used to confirm the dimensions of the questionnaire and the proposed model of exploratory factor analysis. In this study, indices of Chi-square, (RMSEA), (NFI), (GFI), and (AGFI) were evaluated.

## Results

The findings showed that 55% of professors that participated in content validity step were female, 55% were associate professor, 27% were assistant professor and the rest of them were professor. The mean age of the participants was 48.92 ± 4.29. In addition, the average service history of the participants was 17.01 ± 5.08 years.

Of the 211 students that participated in study, 35.5% were nursing and 64.5% were medicine students. Moreover, in the group of nursing students, 32% were males, and 68% were females. On the other hand, in the medicine group, 48% were males, and 52% were females. The mean age of nursing students was 21.74 ± 3.5 while for medicine students was 22.98 ± 2.73.

### Content validity (qualitative and quantitative)

After obtaining qualitative content validity in terms of relevance, comprehensibility, grammar, language, scoring, the main and major aspects of the investigated concept, components and totality of the tool, adequacy of the items, and their clarity and simplicity, quantitative content validity with checking CVR and CVI was held. According to the panel of experts (11 participants), the acceptable CVR according to the Lawshe table was 0.59. Thus, the items whose CVR was above 0.59 were maintained in the questionnaire, and the remaining ones were eliminated. Findings showed that all Items have CVR scores of more than 0.59.

In the next step, the CVI was measured with participating of 11 relevant experts and specialists. The CVI value was determined at 0.76. Findings showed that 38 Items have acceptable CVI value with only four items that needing revision. The overall CVI of the questionnaire was 0.87.

### Construct validity (Exploratory and Confirmatory Factor Analysis)

To determine the sample size adequacy and appropriateness of the factor model, the Kaiser Meyer Olkin Index (KMO) and the Bartlett test of sphericity were calculated. Bartlett’s test of sphericity (X^2^ = 381.527, *P* = 0.001) indicated that the exploratory factor analysis was acceptably carried out. The result of KMO was 0.84 that indicated the total number of samples was sufficient. All Items had communality more than 0.5.

**Fig. 1:**
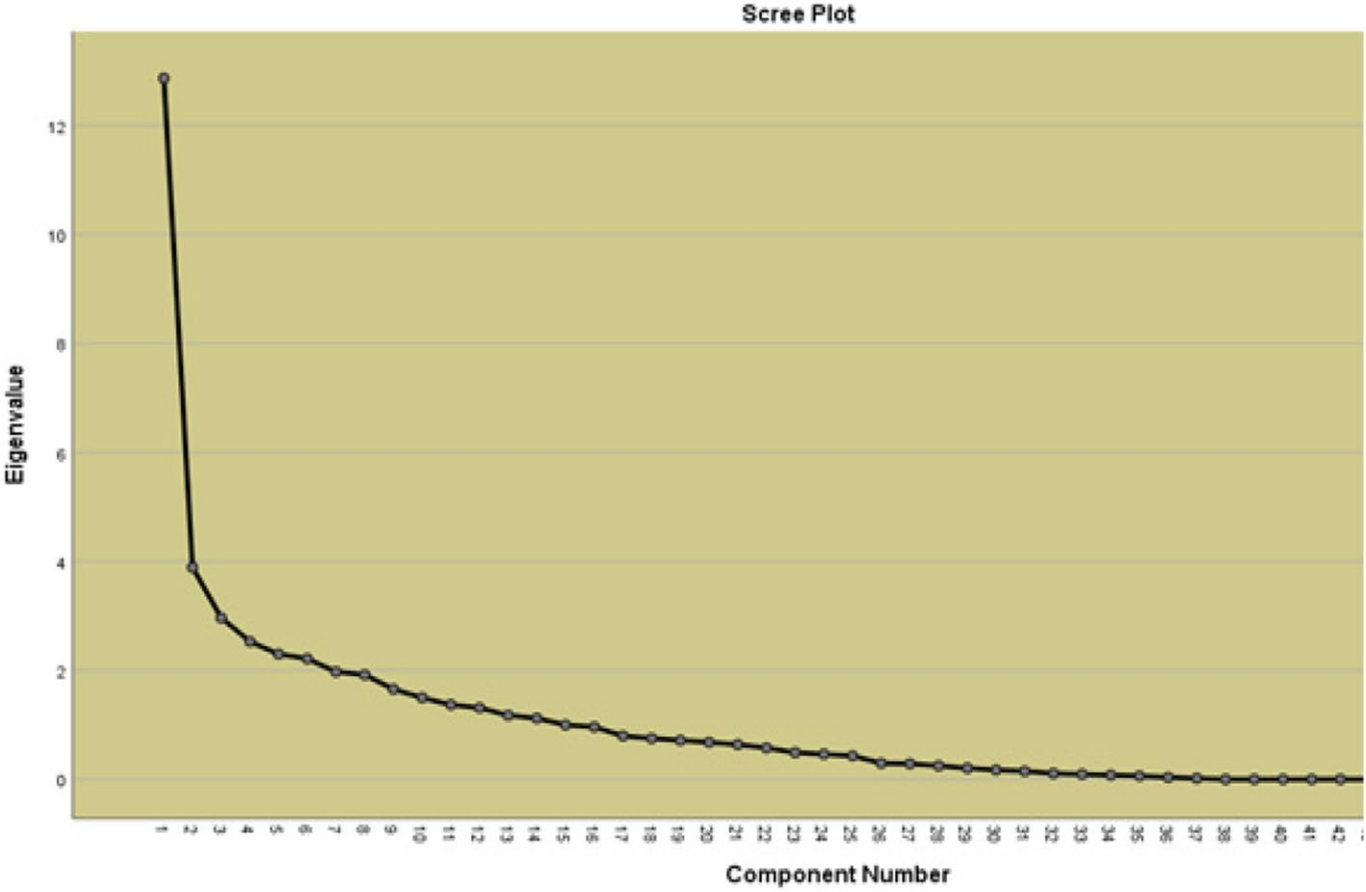
Scree plot

First, exploratory factor analysis with Varimax rotation revealed more than 10 factors have eigenvalues above 1. Parallel analysis to confirm the extracted factors showed that just four factors had real eigenvalue greater than the average value of the parallel analysis, which four factors preserved for exploratory factor analysis. The results are shown in Fig. 1. According to Table 1, four factors with eigenvalues above 1 that explain 52.009% of the total variance.

**Table 1:** The percentage, variance, and eigenvalues of extracted factors

**Total Variance Explained**									
**Component**	Initial Eigenvalues			Extraction Sums of Squared Loadings			Rotation Sums of Squared Loadings		
	Total	% of Variance	Cumulative %	Total	% of Variance	Cumulative %	Total	% of Variance	Cumulative %
**1**	9.770	23.828	23.828	9.770	23.828	23.828	5.699	13.901	13.901
**2**	6.219	15.167	38.995	6.219	15.167	38.995	5.371	13.100	27.001
**3**	5.222	12.737	51.732	5.222	12.737	51.732	5.247	12.798	39.799
**4**	3.338	8.142	59.874	3.338	8.142	59.874	5.006	12.210	52.009

Extraction method: principal component analysis

All items had factor loading more than 0.7 so none of the items were deleted. According to Table 2, all factors had more than 3 items. Based on the results of EFA, factors were named based on the items in each factor, factor 1: values and ethics (10 items), factor 2: roles and responsibilities (9 items), factor 3: inter-professional communication (11 items), and factor 4: teams and teamwork (12 items).

**Table 2:** Principal component analysis with Varimax rotation

*Item*	*First factor*	*Second factor*	*Third factor*	*Fourth factor*
Place patients’ interests at the center of inter-professional healthcare services	871			
Respect patients’ privacy and confidentiality in offering team-based healthcare services	872			
Recognize variety as a characteristic of patients and healthcare teams	.551			
Respect unique cultures, values, roles/responsibilities, and expertise of other healthcare professions	.899			
Cooperate with people who are cared for or provide care and support	.908			
Establish trustworthy relationships with patients, their families, and other members of the team	.901			
Show high ethical and care standards in team-based healthcare	.945			
Manage particular ethical issues that arise in patient-oriented inter-professional healthcare situations	.934			
Act truthfully and conscientiously in relationships with patients, their families, and other members of the team	.935			
Maintain my professional competency in line with the level or area of my work or training	.948			
Explicitly share my roles and responsibilities with patients, their families, and other specialists		.905		
Know my limitations in terms of skills, knowledge, and abilities		.912		
Involve various supplementary professional specialists to create strategies required to meet my patients’ special healthcare needs		.890		
Explain the roles and responsibilities of other healthcare providers and the manner of performing teamwork to provide healthcare		.919		
Implement the knowledge, skills, and capabilities of other healthcare specialists and staff to offer secure, timely, efficient, effective, and fair healthcare		.922		
Communicate with other team members to clarify each person’s responsibility in carrying out a treatment program or a general health intervention		.846		
Establish inter-professional relationships to improve healthcare and develop learning		.947		
Become involved in perpetual professional and inter-professional development to enhance team performance		.924		
Use the unique and supplementary capabilities of all team members to optimize healthcare		.930		
Select effective communication instruments and techniques to facilitate discussions and interactions that improve team performance			.932	
Share information with patients, their families, and other members of healthcare teams in a comprehensible manner			.894	
If possible, refrain from using specialized terminology			.915	
Communicate my knowledge and views to other members of a team in a particular healthcare case with clarity and respect			.877	
Listen actively and encourage other team members’ ideas and opinions			.962	
Provide other team members with timely and sensitive feedback on their performance			.868	
Respond to the feedback provided by other team members with respect			.851	
Use convenient and respectful language in a difficult situation – e.g., inter-professional conflict			.910	
Detect how my experience and expertise can be helpful in communications, conflict resolution, and inter-professional relationships			.833	
Detect how my status in a healthcare team’s hierarchy can be helpful in communications, conflict resolution, and inter-professional relationships			.868	
Permanently highlight the importance of group work in patient- and society-oriented healthcare			.710	
Explain the process of team development				954
Explain the roles and actions of effective healthcare teams				.924
Involve other healthcare professions in solving common problems according to a particular healthcare situation				.938
Make healthcare decisions by incorporating the knowledge and experience of other professions according to a particular healthcare situation				.927
Use leadership methods that support participatory work and team effectiveness				.895
Involve others in the constructive management of conflicts between various healthcare professions, patients, and their families				.919
Share responsibility with other professions, patients, and communities in terms of prevention and healthcare services				.863
Rethink my performance to improve it				.903
Rethink the performance of my healthcare team to improve it				.923
Use strategies that improve the effectiveness of inter-professional teamwork and team-based healthcare				.880
Use the existing evidence to get informed about effective teamwork and team-based methods				.903
Act effectively in various teams and roles across different environments				.836

To check the construct and confirm the dimensions of the questionnaire, confirmatory factor analysis with PLS was used. To confirm the homogeneity of the items in terms of the content and face validity of the detected dimensions, a four-factor confirmatory factor analysis was performed on the questionnaires. The AVE value of the coefficient varies from 0 to 1, with values above 0.5 as acceptable ones. Moreover, the CR value ranges between 0 and 1, with values above 0.7 considered acceptable and values below 0.6 as unpeaceable; investigating the inter-construct index showed that the obtained value for the coefficient was above the determined threshold across all constructs in the study, and, thus, their convergent validity was at an acceptable level (Table 3).

**Table 3:** Convergent validity, composite reliability

**Dimension**	AVE	CR	t-value
Values and ethics	0.76	0.91	6.32
Roles and responsibilities	0.68	0.85	8.24
Inter-professional communication	0.64	0.91	5.29
team-based care and teamwork	0.72	0.89	6.42

The values of fit indices indicated the appropriate fit of the four dimensions model (Table 4). All indicators and components had factor loading above 0.7; therefore, the membership of all investigated factors has been confirmed (Fig. 1).

**Table 4:** Model fit of IPEC questionnaire

Index	Standard	The value of the index in the intended model
Relative chi-square	At most between 2 and 3	2.38
RMSE	Below 0.1	0.026
NFI	Above 0.91	0.93
NNFI	Above 0.9	0.95
CFI	Above 0.9	0.92
RFI	Above 0.9	0.91
IFI	Above 0.9	0.93
GFI	Equal to or above 0.9	0.95

**Fig. 2:**
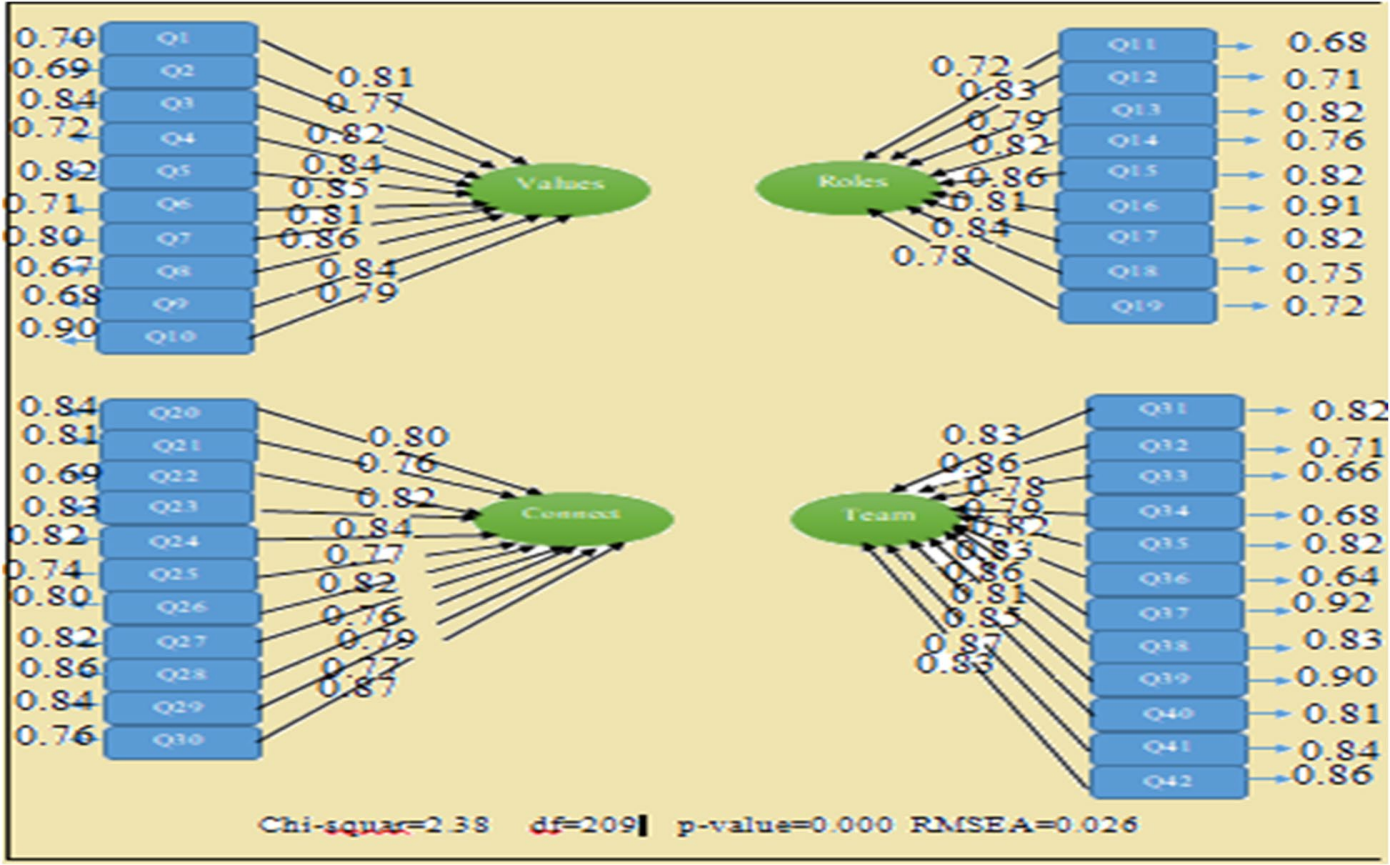
Scores obtained with four dimensions in the standard model

Scores obtained with four dimensions in the standard model (Df, chi-squar, p-value, RMSEA) It shows that the model has the necessary and sufficient validity (Fig. 2).

#### Stage 4: reliability (Internal consistency and Stability)

Cronbach’s Alpha coefficient for total items and All subscales were more than 0.70, that indicated acceptable internal consistency for IPEC questionnaire.

The ICC was 0.82, which is acceptable. This coefficient for dimensions of Values and ethics, Roles and responsibilities, Inter-professional communication, and team-based care and teamwork were 0.83, 0.84, 0.79, and 0.82 respectively (Table 5).

**Table 5:** Cronbach’s Alpha and ICC values for four dimensions and all items

Factor	Scale	Items	Cronbach’s Alpha	Intra-class correlation coefficient
1	Values and ethics	1–10	0.85	0.83
2	Roles and responsibilities	11–19	.077	0.84
3	Inter-professional communication	20–30	0.84	0.79
4	Team-based care and teamwork	31–42	0.89	0.82
Total		42	0.84	0.82

## Discussion

The inter-professional education competence (IPEC) instrument is among the few tools that can be used to self-assess inter-professional competencies as a way of raising awareness of one’s competencies and trying to improve them. Shifting toward inter-professional education is among the major developments in healthcare education. The main and ultimate goal of inter-professional education is to enhance the healthcare services provided to patients. The results of some studies have indicated the positive impacts of inter-professional education on the improved performance of healthcare team members. On the other hand, some other studies have considered such education ineffective, indicating that inter-professional education is more effective in improving knowledge and skills. One of the important concerns in educational systems is the development of learners' skills to achieve patient safety and provide team-oriented services. This is while the students are mainly learning their professional skills in a single profession and in separate faculties. This has caused professional skills to be given special attention and non-technical skills such as inter-professional cooperation skills, communication and karmic skills, etc. do not have a place in single professional training programs [20]. Madineh Jassemi et al. conducted a cross-sectional investigation of the attitudes of nurses and doctors toward inter-professional collaboration and found that doctors had limited views toward inter-professional collaboration, particularly in terms of doctors’ hegemony and nurses’ autonomy. Thus, they recommended the adoption of approaches like shared educational courses for the students of Nursing and medicine to improve their inter-professional collaboration. Jefferson questionnaire was used in this study. This questionnaire was prepared by the researchers of Jefferson University in Philadelphia and Pennsylvania in 2001 to measure the views of nurses and doctors regarding interprofessional cooperation. The above questionnaire has been used in many studies in different countries such as Italy, Mexico, America and Turkey, and its validity and reliability have been confirmed. This questionnaire consists of two parts, the first part contains a list of questions related to the demographic profile of the person participating in the study and the second part has 15 questions based on a four-point Likert scale. The fields of this tool include the mastery of doctors, Nurses' autonomy, teamwork and treatment versus care [21, 22]. Moreover, during a qualitative study in Isfahan, Iran, Irajpour found that nurses evaluated their professional relations with doctors as inappropriate and most of the time argued that doctors lacked any awareness of nurses’ professional duties and roles. Thus, based on the above studies, inter-professional collaboration needs to be assessed from university years and be promoted as a competency among the students of medicine and nursing [14]. The results of Oates' study, which aimed to critically evaluate tools for measuring the ability of interprofessional cooperation, showed that most of the tools in this field are related to measuring attitudes, and limited tools measure the behavior of interprofessional cooperation [23] this questionnaire was different from the psychometric questionnaire in the present study both in dimensions and in the number of items.

(ICAR) Rubric is a tool for measuring interprofessional collaboration behavior. This tool was developed by Curran Vernon and his colleagues in 2011 in Canada. The first version of the ICAR instrument includes 30 items in the areas of communication skills, cooperation, roles and responsibilities, teamwork, challenge management, communication with the patient and his companions. The revision of this tool was done in the 2013 version with the aim of improving the functionality of the tool. 13 items in different areas were removed and the rubric was finalized with 17 items [24]. This questionnaire was different from the psychometric questionnaire in the present study both in dimensions and in the number of items. Stichler collaborative behavior scale (CBS) was compiled with the aim of determining the extent of interprofessional behaviors in doctors and nurses. This tool is developed as a conceptual framework in relation to interactive and social theory and is used for nurses to evaluate behaviors in interprofessional cooperation in mutual situations and also to measure the effects of power sharing in interprofessional cooperation [25, 26]. This questionnaire was different from the psychometric questionnaire in the present study both in dimensions and in the number of items.

## Conclusion

As a result, due to the importance and value of inter-professional collaboration competencies between medical and nursing students, it is necessary to have access to a valid and reliable tool to assess the variable, the present study aimed to conduct a psychometric investigation on this tool so that it could be used in convenient educational-clinical situations. The questionnaire investigated in the present study is a relevant and useful tool with acceptable content validity and reliability and can be implemented to evaluate inter-professional collaboration among medical and nursing students. Using the instrument, students’ attitudes toward inter-professional collaboration can be assessed during their university years, and, in case they have negative attitudes, this can be corrected by planners involved in educational planning to offer the students a more successful profession in the future.

The present study aimed to translate and conduct a psychometric investigation of the inter-professional education competence (IPEC) tool for the students of medicine and nursing. The results showed that the main tool fulfilled acceptable indicators according to a panel of experts. The tools evaluate inter-professional collaboration competency between nursing and medical students across four areas (values and ethics, roles and responsibilities, inter-professional communication, and team-based care and teamwork). Specialists in curriculum development can move toward inter-professional education and development for students by considering the items of the instrument in their educational plans and curriculum development.

Our limitations in conducting this study were the lack of access to all the studied samples at the time of the researcher's visit, which was addressed several times to complete the questionnaires.

## Data Availability

The datasets used and analyzed during the current study available from the corresponding author on reasonable request.
